# Supplementary material for: tRNAscan-SE 2.0: improved detection and functional classification of transfer RNA genes
Source: Nucleic Acids Res. 2021 Aug 20;49(16):9077–96. doi: 10.1093/nar/gkab688 (PMC8450103; doi:10.1093/nar/gkab688)
Supplement: gkab688_Supplemental_Files [file gkab688_supplemental_files.zip › SupplementaryData.pdf]

SUPPLEMENTARY TABLES AND FIGURES

Supplementary Table S1 Data source of specific genomes analyzed in this manuscript.

Genome	Domain	Assembly/Accession
Arabidopsis thaliana	Eukaryota	TAIR10
Armadillo (*Dasypus novemcinctus*)	Eukaryota	Dasnov3.0
Budding yeast (*Saccharomyces cerevisiae* S288C)	Eukaryota	GCA_000146045.2/R64
Caenorhabditis elegans	Eukaryota	WBcel235
Cat (*Felis catus*)	Eukaryota	Felis_catus_9.0
Chicken (*Gallus gallus*)	Eukaryota	GRCg6a
Cow (*Bos taurus*)	Eukaryota	Bos_taurus_UMD_3.1.1
Dolphin (*Tursiops truncates*)	Eukaryota	Ttru_1.4
Elephant shark (*Callorhinchus milii*)	Eukaryota	Callorhinchus_milii-6.1.3
Ferret (*Mustela putorius furo*)	Eukaryota	MusPutFur1.0
Human (*Homo sapiens*)	Eukaryota	GRCh37/hg19 and GRCh38/hg38
Japanese rice (*Oryza sativa* Japonica Group)	Eukaryota	IRGSP-1.0
Maize (*Zea mays*)	Eukaryota	RefGen_v4
Medaka (*Oryzias latipes*)	Eukaryota	GCA_002234675.1/ASM223467v1
Minke whale (*Balaenoptera acutorostrata scammoni*)	Eukaryota	BalAcu1.0
Mouse (*Mus musculus*)	Eukaryota	GRCm38/mm10
Panda (*Ailuropoda melanoleuca*)	Eukaryota	AilMel_1.0
Rat (*Rattus norvegicus*)	Eukaryota	Rnor6.0
Rhesus (*Macaca mulatta*)	Eukaryota	Mmul8.0.1
Sheep (*Ovis aries*)	Eukaryota	Oar_v4.0
Squirrel (*Spermophilus tridecemlineatus*)	Eukaryota	SpeTri2.0
Bacillus subtilis subsp. subtilis str. 168	Bacteria	GCA_000009045.1/ASM904v1
Clostridioides difficile 630	Bacteria	GCA_000009205.2/ASM920v2
Escherichia coli str. K-12 substr. MG1655	Bacteria	GCA_000005845.2/ASM584v2
Gloeobacter violaceus PCC 7421	Bacteria	GCA_000011385.1/ ASM1138v1
Mycobacterium tuberculosis H37Rv	Bacteria	GCA_000195955.2/ASM19595v2
Pseudomonas aeruginosa PAO1	Bacteria	GCA_000006765.1/ASM676v1
Synechocystis sp. PCC 6803	Bacteria	GCA_000009725.1/ ASM972v1
Halobacterium sp. NRC-1	Archaea	GCA_000006805.1/ASM680v1
Methanobrevibacter ruminantium M1	Archaea	GCA_000024185.1/ASM2418v1
Methanobrevibacter smittii ATCC 35061	Archaea	GCA_000016525.1/ASM1652v1
Methanobrevibacter sp. AbM4	Archaea	GCA_000404165.1/ASM40416v1
Methanocaldococcus jannaschii DSM 2661	Archaea	GCA_000091665.1/ASM9166v1
Pyrobaculum aerophilum IM2	Archaea	GCA_000007225.1/ASM722v1
Pyrobaculum calidifontis JCM 11548	Archaea	GCA_000015805.1/ASM1580v1
Pyrococcus furiosus DSM 3638	Archaea	GCA_000007305.1/ASM730v1
Sulfolobus acidocaldarius DSM 639	Archaea	GCA_000012285.1/ASM1228v1
Chirocentrus dorab mitochondrion	Eukaryota	NC_006913.1
Desmognathus fuscus mitochondrion	Eukaryota	NC_006339.1
Diretmus argenteus mitochondrion	Eukaryota	NC_008127.1
Hemiechinus auritus mitochondrion	Eukaryota	NC_005033.1
Homo sapiens mitochondrion	Eukaryota	NC_012920.1
Solenostomus cyanopterus mitochondrion	Eukaryota	NC_010267.1
Taeniopygia guttata mitochondrion	Eukaryota	NC_007897.1

Supplementary Table S2 Covariance models used in tRNAscan-SE for (A) default search, (B) legacy search, and (C) mitochondrial search. All covariance models except HMM (hidden Markov model) models include both primary sequence and secondary structure information. "HMM models" used in this study are actually covariance models that were built with only primary sequence information, and can be used as HMMs. Options in parentheses under "Search mode" represent the tRNAscan-SE options used for specific types of searches. Domain-specific covariance models, $tRNA^{SeC}$ models, and mitochondrial tRNA (mt-tRNA) models are listed by their file names (*.cm) under "Covariance model". Isotype-specific covariance models for archaeal, bacterial, and eukaryotic search modes are grouped in these covariance model databases, respectively, with the model names listed under "Covariance model" and the database file names in parentheses. Covariance models were built by Infernal (1) for (A) and (C), and by Cove (3) for (B).

A

Search mode	Covariance model	Description
Archaeal (option -A)	TRNAinf-arch.cm	Archaeal tRNA-specific model
	TRNAinf-arch-ns.cm	Archaeal tRNA-specific HMM model
	arch-Ala (TRNAinf-arch-iso)	Archaeal $tRNA^{Ala}$ model
	arch-Arg (TRNAinf-arch-iso)	Archaeal $tRNA^{Arg}$ model
	arch-Asn (TRNAinf-arch-iso)	Archaeal $tRNA^{Asn}$ model
	arch-Asp (TRNAinf-arch-iso)	Archaeal $tRNA^{Asp}$ model
	arch-Cys (TRNAinf-arch-iso)	Archaeal $tRNA^{Cys}$ model
	arch-Gln (TRNAinf-arch-iso)	Archaeal $tRNA^{Gln}$ model
	arch-Glu (TRNAinf-arch-iso)	Archaeal $tRNA^{Glu}$ model
	arch-Gly (TRNAinf-arch-iso)	Archaeal $tRNA^{Gly}$ model
	arch-His (TRNAinf-arch-iso)	Archaeal $tRNA^{His}$ model
	arch-Ile (TRNAinf-arch-iso)	Archaeal $tRNA^{Ile}$ model
	arch-Ile2 (TRNAinf-arch-iso)	Archaeal $tRNA^{Ile2}$ model
	arch-iMet (TRNAinf-arch-iso)	Archaeal $tRNA^{iMet}$ model
	arch-Leu (TRNAinf-arch-iso)	Archaeal $tRNA^{Leu}$ model
	arch-Lys (TRNAinf-arch-iso)	Archaeal $tRNA^{Lys}$ model
	arch-Met (TRNAinf-arch-iso)	Archaeal $tRNA^{Met}$ model
	arch-Phe (TRNAinf-arch-iso)	Archaeal $tRNA^{Phe}$ model
	arch-Pro (TRNAinf-arch-iso)	Archaeal $tRNA^{Pro}$ model
	arch-Ser (TRNAinf-arch-iso)	Archaeal $tRNA^{Ser}$ model
	arch-Thr (TRNAinf-arch-iso)	Archaeal $tRNA^{Thr}$ model
	arch-Trp (TRNAinf-arch-iso)	Archaeal $tRNA^{Trp}$ model
	arch-Tyr (TRNAinf-arch-iso)	Archaeal $tRNA^{Tyr}$ model
	arch-Val (TRNAinf-arch-iso)	Archaeal $tRNA^{Val}$ model
	TRNAinf-arch-SeC.cm	Archaeal $tRNA^{SeC}$ model
	Cren-eury-BHB-noncan.cm	Model of noncanonical introns in Crenarchaeaota and Euryarchaeota
	Thaum-BHB-noncan.cm	Model of noncanonical introns in Thaumarchaeaota
Bacterial (option -B)	TRNAinf-bact.cm	Bacterial tRNA-specific model
	TRNAinf-bact-ns.cm	Bacterial tRNA-specific HMM model
	bact-Ala (TRNAinf-bact-iso)	Bacterial $tRNA^{Ala}$ model
	bact-Arg (TRNAinf-bact-iso)	Bacterial $tRNA^{Arg}$ model
	bact-Asn (TRNAinf-bact-iso)	Bacterial $tRNA^{Asn}$ model
	bact-Asp (TRNAinf-bact-iso)	Bacterial $tRNA^{Asp}$ model
	bact-Cys (TRNAinf-bact-iso)	Bacterial $tRNA^{Cys}$ model
	bact-Gln (TRNAinf-bact-iso)	Bacterial $tRNA^{Gln}$ model
	bact-Glu (TRNAinf-bact-iso)	Bacterial $tRNA^{Glu}$ model
	bact-Gly (TRNAinf-bact-iso)	Bacterial $tRNA^{Gly}$ model
	bact-His (TRNAinf-bact-iso)	Bacterial $tRNA^{His}$ model

Search mode	Covariance model	Description
	bact-Ile (TRNAinf-bact-iso)	Bacterial $tRNA^{Ile}$ model
	bact-Ile2 (TRNAinf-bact-iso)	Bacterial $tRNA^{Ile2}$ model
	bact-fMet (TRNAinf-bact-iso)	Bacterial $tRNA^{iMet}$ model
	bact-Leu (TRNAinf-bact-iso)	Bacterial $tRNA^{Leu}$ model
	bact-Lys (TRNAinf-bact-iso)	Bacterial $tRNA^{Lys}$ model
	bact-Met (TRNAinf-bact-iso)	Bacterial $tRNA^{Met}$ model
	bact-Phe (TRNAinf-bact-iso)	Bacterial $tRNA^{Phe}$ model
	bact-Pro (TRNAinf-bact-iso)	Bacterial $tRNA^{Pro}$ model
	bact-Ser (TRNAinf-bact-iso)	Bacterial $tRNA^{Ser}$ model
	bact-Thr (TRNAinf-bact-iso)	Bacterial $tRNA^{Thr}$ model
	bact-Trp (TRNAinf-bact-iso)	Bacterial $tRNA^{Trp}$ model
	bact-Tyr (TRNAinf-bact-iso)	Bacterial $tRNA^{Tyr}$ model
	bact-Val (TRNAinf-bact-iso)	Bacterial $tRNA^{Val}$ model
	TRNAinf-bact-SeC.cm	Bacterial $tRNA^{SeC}$ model
Eukaryotic (option -E)	TRNAinf-euk.cm	Eukaryotic tRNA-specific model
	TRNAinf-euk-ns.cm	Eukaryotic tRNA-specific HMM model
	euk-Ala (TRNAinf-euk-iso)	Eukaryotic $tRNA^{Ala}$ model
	euk-Arg (TRNAinf-euk-iso)	Eukaryotic $tRNA^{Arg}$ model
	euk-Asn (TRNAinf-euk-iso)	Eukaryotic $tRNA^{Asn}$ model
	euk-Asp (TRNAinf-euk-iso)	Eukaryotic $tRNA^{Asp}$ model
	euk-Cys (TRNAinf-euk-iso)	Eukaryotic $tRNA^{Cys}$ model
	euk-Gln (TRNAinf-euk-iso)	Eukaryotic $tRNA^{Gln}$ model
	euk-Glu (TRNAinf-euk-iso)	Eukaryotic $tRNA^{Glu}$ model
	euk-Gly (TRNAinf-euk-iso)	Eukaryotic $tRNA^{Gly}$ model
	euk-His (TRNAinf-euk-iso)	Eukaryotic $tRNA^{His}$ model
	euk-Ile (TRNAinf-euk-iso)	Eukaryotic $tRNA^{Ile}$ model
	euk-iMet (TRNAinf-euk-iso)	Eukaryotic $tRNA^{iMet}$ model
	euk-Leu (TRNAinf-euk-iso)	Eukaryotic $tRNA^{Leu}$ model
	euk-Lys (TRNAinf-euk-iso)	Eukaryotic $tRNA^{Lys}$ model
	euk-Met (TRNAinf-euk-iso)	Eukaryotic $tRNA^{Met}$ model
	euk-Phe (TRNAinf-euk-iso)	Eukaryotic $tRNA^{Phe}$ model
	euk-Pro (TRNAinf-euk-iso)	Eukaryotic $tRNA^{Pro}$ model
	euk-Ser (TRNAinf-euk-iso)	Eukaryotic $tRNA^{Ser}$ model
	euk-Thr (TRNAinf-euk-iso)	Eukaryotic $tRNA^{Thr}$ model
	euk-Trp (TRNAinf-euk-iso)	Eukaryotic $tRNA^{Trp}$ model
	euk-Tyr (TRNAinf-euk-iso)	Eukaryotic $tRNA^{Tyr}$ model
	euk-Val (TRNAinf-euk-iso)	Eukaryotic $tRNA^{Val}$ model
	TRNAinf-euk-SeC.cm	Eukaryotic $tRNA^{SeC}$ model
General (option -G)	TRNAinf.cm	General tRNA model
	TRNAinf-ns.cm	General tRNA HMM model
Organellar (option -O)	TRNAinf-1415.cm	General tRNA model trained with original Sprinzl database (2)
	TRNAinf-1415-ns.cm	General tRNA HMM model trained with original Sprinzl database (2)

B

Search mode	Covariance model	Description
Archaeal (option -L -A)	TRNA2-arch.cm	Archaeal tRNA-specific model
	TRNA2-archns.cm	Archaeal tRNA-specific HMM model
	PSELC.cm	Prokaryotic $tRNA^{SeC}$ model
Bacterial (option -L -B)	TRNA2-bact.cm	Bacterial tRNA-specific model
	TRNA2-bactns.cm	Bacterial tRNA-specific HMM model
	PSELC.cm	Prokaryotic $tRNA^{SeC}$ model

Search mode	Covariance model	Description
Eukaryotic (option -L -E)	TRNA2-euk.cm	Eukaryotic tRNA-specific model
	TRNA2-eukns.cm	Eukaryotic tRNA-specific HMM model
	ESELC.cm	Eukaryotic tRNA^{SeC} model
General (option -L -G)	TRNA2.cm	General tRNA model
	TRNA2ns.cm	General tRNA HMM model
Organellar (option -C -O)	TRNA2.cm	General tRNA model
	TRNA2ns.cm	General tRNA HMM model

C

Search mode	Covariance model	Description
Mammalian mt-tRNA (option -M mammal)<br><br>Cytosolic/mito tRNA detemination – use with eukaryotic search mode (option --mt mammal)	TRNAinf-mito-mammal-Ala.cm	Mammalian mt-tRNA^{Ala} model
	TRNAinf-mito-mammal-Arg.cm	Mammalian mt-tRNA^{Arg} model
	TRNAinf-mito-mammal-Asn.cm	Mammalian mt-tRNA^{Asn} model
	TRNAinf-mito-mammal-Asp.cm	Mammalian mt-tRNA^{Asp} model
	TRNAinf-mito-mammal-Cys.cm	Mammalian mt-tRNA^{Cys} model
	TRNAinf-mito-mammal-Gln.cm	Mammalian mt-tRNA^{Gln} model
	TRNAinf-mito-mammal-Glu.cm	Mammalian mt-tRNA^{Glu} model
	TRNAinf-mito-mammal-Gly.cm	Mammalian mt-tRNA^{Gly} model
	TRNAinf-mito-mammal-His.cm	Mammalian mt-tRNA^{His} model
	TRNAinf-mito-mammal-Ile.cm	Mammalian mt-tRNA^{Ile} model
	TRNAinf-mito-mammal-LeuTAA.cm	Mammalian mt-tRNA$^{Leu(TAA)}$ model
	TRNAinf-mito-mammal-LeuTAG.cm	Mammalian mt-tRNA$^{Leu(TAG)}$ model
	TRNAinf-mito-mammal-Lys.cm	Mammalian mt-tRNA^{Lys} model
	TRNAinf-mito-mammal-Met.cm	Mammalian mt-tRNA^{Met} model
	TRNAinf-mito-mammal-Phe.cm	Mammalian mt-tRNA^{Phe} model
	TRNAinf-mito-mammal-Pro.cm	Mammalian mt-tRNA^{Pro} model
	TRNAinf-mito-mammal-SerGCT.cm	Mammalian mt-tRNA$^{Ser(GCT)}$ model
	TRNAinf-mito-mammal-SerTGA.cm	Mammalian mt-tRNA$^{Ser(TGA)}$ model
	TRNAinf-mito-mammal-Thr.cm	Mammalian mt-tRNA^{Thr} model
	TRNAinf-mito-mammal-Trp.cm	Mammalian mt-tRNA^{Trp} model
	TRNAinf-mito-mammal-Tyr.cm	Mammalian mt-tRNA^{Tyr} model
	TRNAinf-mito-mammal-Val.cm	Mammalian mt-tRNA^{Val} model
Vertebrate mt-tRNA (option -M vert)<br><br>Cytosolic/mito tRNA detemination – use with eukaryotic search mode (option --mt vert)	TRNAinf-mito-vert-Ala.cm	Vertebrate mt-tRNA^{Ala} model
	TRNAinf-mito-vert-Arg.cm	Vertebrate mt-tRNA^{Arg} model
	TRNAinf-mito-vert-Asn.cm	Vertebrate mt-tRNA^{Asn} model
	TRNAinf-mito-vert-Asp.cm	Vertebrate mt-tRNA^{Asp} model
	TRNAinf-mito-vert-Cys.cm	Vertebrate mt-tRNA^{Cys} model
	TRNAinf-mito-vert-Gln.cm	Vertebrate mt-tRNA^{Gln} model
	TRNAinf-mito-vert-Glu.cm	Vertebrate mt-tRNA^{Glu} model
	TRNAinf-mito-vert-Gly.cm	Vertebrate mt-tRNA^{Gly} model
	TRNAinf-mito-vert-His.cm	Vertebrate mt-tRNA^{His} model
	TRNAinf-mito-vert-Ile.cm	Vertebrate mt-tRNA^{Ile} model
	TRNAinf-mito-vert-LeuTAA.cm	Vertebrate mt-tRNA$^{Leu(TAA)}$ model
	TRNAinf-mito-vert-LeuTAG.cm	Vertebrate mt-tRNA$^{Leu(TAG)}$ model
	TRNAinf-mito-vert-Lys.cm	Vertebrate mt-tRNA^{Lys} model
	TRNAinf-mito-vert-Met.cm	Vertebrate mt-tRNA^{Met} model
	TRNAinf-mito-vert-Phe.cm	Vertebrate mt-tRNA^{Phe} model
	TRNAinf-mito-vert-Pro.cm	Vertebrate mt-tRNA^{Pro} model
	TRNAinf-mito-vert-SerGCT.cm	Vertebrate mt-tRNA$^{Ser(GCT)}$ model
	TRNAinf-mito-vert-SerTGA.cm	Vertebrate mt-tRNA$^{Ser(TGA)}$ model
	TRNAinf-mito-vert-Thr.cm	Vertebrate mt-tRNA^{Thr} model
	TRNAinf-mito-vert-Trp.cm	Vertebrate mt-tRNA^{Trp} model
	TRNAinf-mito-vert-Tyr.cm	Vertebrate mt-tRNA^{Tyr} model

Search mode	Covariance model	Description
	TRNAinf-mito-vert-Val.cm	Vertebrate mt-$tRNA^{Val}$ model

Supplementary Table S3 tRNAscan-SE performance comparison for non-default "maximum sensitivity" mode. Nine genomes representing different clades were benchmarked on COVE-only and maximum sensitivity search mode using tRNAscan-SE 1.3 and new version 2.0 respectively. The genome scans using tRNAscan-SE 2.0 were conducted with Infernal (1) running eight threads in parallel. (A) Total number of tRNA predictions and the number of predictions excluding those determined as possible pseudogenes in the nuclear genomes are listed. (B) Reported search times are based on using a server with dual Intel 10-core HT processors at 2.30GHz. tRNAscan-SE 1.3 only allows processing with a single thread while tRNAscan-SE 2.0 makes use of the multi-core processor architecture for parallel computation. *The processing time using tRNAscan-SE 1.3 COVE-only mode for human, mouse, and maize was estimated with the result processing time of chromosomes 17, 13, and 10 in respective genomes. The total number of tRNA predictions for these genomes are therefore not available (NA).

A

Genome	Domain	tRNAscan-SE 1.3		tRNAscan-SE 2.0	
		Total tRNA predictions	tRNAs excluding pseudogenes	Total tRNA predictions	tRNAs excluding pseudogenes
Escherichia coli K-12	Bacteria	88	87	90	88
Pyrococcus furiosis DSM 3638	Archaea	46	46	46	46
Saccharomyces cerevisiae	Eukaryota	275	275	275	275
Homo sapiens	Eukaryota	NA	NA	626	516
Mus musculus	Eukaryota	NA	NA	41,942	4,165
Caenorhabditis elegans	Eukaryota	837	620	724	618
Drosophila melanogaster	Eukaryota	297	292	295	292
Arabidopsis thaliana	Eukaryota	648	635	643	633
Zea mays	Eukaryota	NA	NA	2,103	1,392

B

Genome	Domain	tRNAscan-SE 1.3	tRNAscan-SE 2.0
Escherichia coli K-12	Bacteria	9h 33m	15m 18s
Pyrococcus furiosis DSM 3638	Archaea	3h 52m	14m 43s
Saccharomyces cerevisiae	Eukaryota	1d 1h	1h 1m
Homo sapiens	Eukaryota	94d 12h*	5d
Mus musculus	Eukaryota	175d 19h*	5d 9h
Caenorhabditis elegans	Eukaryota	7d 3h	5h 13m
Drosophila melanogaster	Eukaryota	9d 11h	9h 18m
Arabidopsis thaliana	Eukaryota	8d 12h	6h 4m
Zea mays	Eukaryota	> 110d*	4d 12h

Supplementary Table S4 tRNA predictions for virtual genomes. Virtual genomes were generated using a 5th order Markov chain trained from the original genome. tRNAscan-SE 2.0 default mode with a score cutoff of 10 bits (10 bits below default cutoff) was used to scan the virtual genomes. The number of tRNA hits represents the predictions identified in all virtual genomes of the original one that score 10 bits or above.

Genome	Domain	GC content	# of Virtual Genomes	# of tRNA Hits	Score Range (bits)
Escherichia coli K-12	Bacteria	50.8%	100	37	10.0 – 16.2
Halobacterium sp. NRC-1	Archaea	67.9%	100	39	10.0 – 19.8
Saccharomyces cerevisiae S288C	Eukaryota	38.4%	100	1	16.9
Homo sapiens	Eukaryota	42.4%	100	610<br>17	10.0 – 19.8<br>20.2 – 29.5

Supplementary Table S5 Mitochondrial tRNA predictions for virtual genomes. tRNAscan-SE 2.0 mitochondrial mode with score cutoff set to 0 bits (20 bits below default cutoff) was used to scan the virtual genomes. The number of tRNAscan-SE hits represents the predictions identified in all virtual genomes that scored 0 bits or above. The number of tRNA hits from ARWEN include total predictions across all virtual genomes with predicted non-pseudogenes in parentheses. MiTFi (4) default search mode was used, showing predictions identified in all virtual genomes, with none more significant than the suggested e-value cutoff (0.0001) in parentheses.

Mitochondrial Genome	GC content	# of Virtual Genomes	tRNAscan-SE 2.0		ARWEN	MiTFi
			# of tRNA Hits	Score Range (bits)	# of tRNA Hits (non-pseudogenes)	# of tRNA Hits (better than e-value cutoff)
Homo sapiens	44.4%	3,500	196	3.0 – 8.4	2,679 (1,245)	3 (0)
Hemiechinus auritus	30.9%	3,500	275	0.3 – 16.5	3,908 (2,077)	4 (0)
Chirocentrus dorab	53.2%	3,500	81	0.0 – 6.2	8,241 (6,344)	0 (0)
Desmognathus fuscus	31.0%	3,500	624	1.2 – 9.8	12,096 (7,320)	1 (0)
Taeniopygia guttata	45.9%	3,500	59	0.4 – 5.5	7,712 (5,701)	0 (0)

Supplementary Table S6 Published human RNA-seq abundance for tRNAs predicted by one or both versions of tRNAscan-SE for human genome assembly GRCh37/hg19. (A) Predictions, excluding pseudogenes, only reported by tRNAscan-SE 1.3 and (B) predictions only reported by tRNAscan-SE 2.0 (default search mode) show very low or no detectable abundance based on prior small RNA sequencing studies employing ARM-Seq (5), DM-tRNA-seq (6), or collected by the database DASHR v2 (7), shown in reads per million (RPM). ARM-Seq and DM-tRNA-seq data were processed by using tRAX (https://github.com/UCSC-LoweLab/tRAX) as described in ARM-Seq publication (4). RPM was calculated as (raw read count aligned to a transcript / total number of alignments) x 1,000,000. The total number of alignments instead of unique reads was used in order to allow multiple mappings per read, as there are frequently multiple identical tRNA gene copies in the genome. The highest RPMs of the tissues/conditions in each study were reported. For comparison, (C) tRNA transcripts with data from all three studies predicted by both tRNAscan-SE 1.3 and 2.0, show clear, consistent evidence of transcription. RPM values above the minimum cutoff of 5.0 highlighted in bold.

A

GtRNAdb ID	Locus	tRNAscan-SE 1.3 Score	ARM-Seq RPM	DM-tRNA-seq RPM	DASHR v2 RPM
tRNA-Asp-GTC-7-1	chr1:161501915-161501986 (+)	34.1	3.03	0.22	**29.91**
tRNA-Gln-CTG-15-1	chr9:126655522-126655594 (-)	23.0	0.00	0.00	0.58
tRNA-Gln-CTG-18-1	chr16:71724890-71724963 (+)	20.8	0.00	0.00	0.01
tRNA-Glu-CTC-17-1	chr1:149334272-149334340 (+)	21.6	0.09	0.00	4.19
tRNA-Glu-CTC-6-1	chr18:43299751-43299822 (+)	32.1	**81.65**	**30.82**	1.27
tRNA-Glu-CTC-8-1	chr3:125413177-125413248 (-)	29.9	0.15	0.20	2.97
tRNA-Glu-TTC-16-1	chr1:149719802-149719870 (+)	21.3	0.09	0.00	4.19
tRNA-His-GTG-3-1	chr3:148316563-148316634 (-)	22.5	0.25	0.00	0.91
tRNA-Leu-CAG-3-1	chr5:159392041-159392118 (-)	20.5	0.00	0.00	0.00
tRNA-Lys-TTT-16-1	chr19:19852207-19852277 (+)	28.3	0.34	0.31	2.23
tRNA-Sup-CTA-1-1	chr17:15408685-15408758 (+)	28.0	0.00	0.00	0.01
tRNA-Sup-TTA-2-1	chr21:15926516-15926586 (+)	22.4	0.00	0.00	0.00
tRNA-Trp-CCA-6-1	chr9:115616989-115617087 (+)	23.4	0.00	0.00	0.40
tRNA-Trp-CCA-7-1	chr11:45290200-45290273 (-)	21.2	0.00	0.00	0.01
tRNA-Val-CAC-12-1	chr6:27650488-27650561 (+)	33.4	0.14	0.10	1.05

B

GtRNAdb ID	Locus	tRNAscan-SE 2.0 Score	ARM-Seq RPM	DM-tRNA-seq RPM	DASHR v2 RPM
tRX-Ala-NNN-1-1	chr6:26713898-26713970 (+)	24.8	0.00	2.33	0.12
tRX-Ala-NNN-1-2	chr6:26788156-26788228 (-)	24.8	0.00	2.33	0.12
tRX-Ala-NNN-2-1	chr6:58156450-58156522 (-)	26.3	0.00	2.33	0.12
tRX-Ala-NNN-3-1	chr19:12299376-12299449 (+)	37.8	1.36	0.14	0.04
tRX-Ala-NNN-4-1	chr16:80479452-80479528 (-)	38.2	0.00	0.00	0.23
tRX-Asn-NNN-1-1	chr1:147789485-147789554 (-)	20.4	0.00	0.00	**400.0**
tRX-Asp-NNN-1-1	chr6:28795190-28795261 (-)	34.9	**19.03**	3.62	**12.27**
tRX-Cys-NNN-1-1	chr7:149103155-149103226 (-)	34.0	0.00	0.06	0.05
tRX-Cys-NNN-2-1	chr8:9939323-9939393 (-)	27.2	2.52	0.07	4.38
tRX-Cys-NNN-4-1	chr7:149325244-149325326 (+)	26.6	3.39	0.00	**136.8**
tRX-Gln-NNN-1-1	chr8:89145236-89145309 (-)	21.5	0.00	0.00	0.00
tRX-Glu-NNN-1-1	chr8:11770800-11770871 (+)	21.9	**45.29**	1.27	**96.75**
tRX-Ile-NNN-1-1	chrX:3833685-3833758 (-)	46.5	0.00	0.00	0.03
tRX-Ile-NNN-1-2	chrX:3795256-3795329 (-)	46.5	0.00	0.00	0.03

GtRNAdb ID	Locus	tRNAscan-SE 2.0 Score	ARM-Seq RPM	DM-tRNA-seq RPM	DASHR v2 RPM
tRX-Ile-NNN-3-1	chr6:27228710-27228784 (+)	32.4	0.00	0.00	4.07
tRX-Leu-NNN-2-1	chr11:113432995-113433078 (-)	45.5	0.00	0.28	0.24
tRX-Leu-NNN-3-1	chr2:152687652-152687719 (-)	22.0	0.00	0.00	0.00
tRX-Lys-NNN-1-1	chr16:2977662-2977734 (+)	46.8	0.85	0.50	**33.70**
tRX-Lys-NNN-2-1	chr3:15252973-15253048 (+)	41.4	4.04	1.50	**55.81**
tRX-Lys-NNN-6-1	chr5:167816644-167816715 (-)	29.9	0.00	0.17	4.48
tRX-Ser-NNN-2-1	chrX:25280089-25280160 (-)	21.6	0.00	0.00	0.00
tRX-Tyr-NNN-1-1	chr14:21137333-21137418 (-)	22.8	**6.89**	0.12	**7.93**
tRX-Val-NNN-1-1	chr6:158824648-158824720 (-)	33.8	**555.85**	**85.85**	**34.54**
tRX-Val-NNN-2-1	chr2:85085876-85085948 (+)	47.6	**6.94**	0.26	**6.12**

C

GtRNAdb Transcript ID	ARM-Seq RPM	DM-tRNA-seq RPM	DASHR v2 RPM
tRNA-Arg-ACG-1	**2516.19**	**3920.81**	**331.43**
tRNA-Arg-CCG-2	**197.66**	**1687.58**	**8602.75**
tRNA-Arg-CCT-4	**140.86**	**697.99**	**1570.98**
tRNA-Arg-TCG-3	**353.59**	**2747.50**	**942.71**
tRNA-Arg-TCG-5	**107.45**	**1353.45**	**76.28**
tRNA-Arg-TCT-1	**166.02**	**761.73**	**3335.3**
tRNA-Asn-GTT-3	**483.26**	**7733.13**	**353.23**
tRNA-Gln-CTG-1	**8811.83**	**30231.34**	**546.83**
tRNA-Gln-TTG-1	**839.62**	**1822.19**	**331.83**
tRNA-Gly-CCC-2	**854.22**	**6031.06**	**19157.94**
tRNA-His-GTG-1	**47604.36**	**47683.55**	**4234.54**
tRNA-iMet-CAT-1	**4574.68**	**48294.91**	**953.67**
tRNA-Leu-AAG-1	**388.43**	**1448.88**	**104.26**
tRNA-Leu-AAG-2	**689.97**	**2625.32**	**244.66**
tRNA-Leu-CAA-1	**436.53**	**7708.91**	**59.73**
tRNA-Leu-CAA-3	**126.11**	**1583.25**	**69.99**
tRNA-Leu-CAA-4	**316.77**	**1894.11**	**693.69**
tRNA-Leu-CAG-1	**1073.59**	**16999.12**	**377.06**
tRNA-Leu-CAG-1	**889.63**	**16999.12**	**377.06**
tRNA-Leu-CAG-2	**368.80**	**4271.44**	**374.48**
tRNA-Leu-TAA-1	**48.00**	**1570.88**	**1647.96**
tRNA-Leu-TAA-3	4.79	**677.20**	**277.74**
tRNA-Leu-TAG-1	**130.93**	**565.19**	**121.69**
tRNA-Leu-TAG-3	**124.25**	**519.45**	**806.86**
tRNA-Lys-CTT-1	**1169.83**	**3386.12**	**29182.64**
tRNA-Met-CAT-1	**113.52**	**662.97**	**1276.43**
tRNA-Met-CAT-6	**177.66**	**973.06**	**903.79**
tRNA-Phe-GAA-1	**1116.33**	**23229.68**	**428.89**
tRNA-Pro-TGG-3	**5872.17**	**14288.90**	**195.43**
tRNA-Ser-AGA-2	**3985.58**	**17109.11**	**527.92**
tRNA-Ser-GCT-6	**332.23**	**1366.31**	**1328.29**
tRNA-Ser-TGA-3	**673.52**	**3012.75**	**526.39**
tRNA-Thr-TGT-4	**338.12**	**1034.45**	**250.59**
tRNA-Val-AAC-1	**3917.56**	**65305.70**	**5175.19**

Supplementary Table S7 Archaeal tRNA intron predictions by clade, type, and count per gene.

Clade	# of Genomes	# of tRNA Genes	# of Canonical Introns	# of Non-canonical Introns	# of Introns Per tRNA Gene			
					0	1	2	3
Archaea (total)	217	10372	1533	673	8402	1750	204	16
Crenarchaeota	60	2814	914	531	1553	1093	152	16
Acidilobales	2	92	16	9	67	25	0	0
Desulfurococcales	13	609	181	38	399	201	9	0
Fervidicoccales	1	45	8	7	33	9	3	0
Sulfolobales	29	1369	521	78	793	553	23	0
Thermoproteales	15	699	188	399	261	305	117	16
Euryarchaeota	144	6970	520	25	6432	531	7	0
DHVE2 group	2	92	31	0	61	31	0	0
Archaeoglobales	7	339	27	0	312	27	0	0
Halobacteriales	30	1481	106	0	1375	106	0	0
Methanobacteriales	13	559	35	0	524	35	0	0
Methanocellales	3	154	13	4	137	17	0	0
Methanococcales	16	605	32	0	573	32	0	0
Methanomicrobiales	9	449	26	0	423	26	0	0
Methanopyrales	1	35	8	2	27	6	2	0
Methanosarcinales	35	1958	172	11	1778	177	3	0
Thermococcales	20	929	40	0	889	40	0	0
Methanomassiliicoccales	3	138	13	6	120	17	1	0
Thermoplasmatales	5	231	17	1	213	18	0	0
Korarchaeota	1	46	3	3	41	4	1	0
Nanoarchaeota	1	44	3	1	40	4	0	0
Thaumarchaeota	11	498	93	107	337	122	39	0
Cenarchaeales	1	46	6	8	35	8	3	0
Nitrosopumilales	5	218	27	29	169	42	7	0
Nitrososphaerales	3	141	44	53	66	53	22	0
unclassified	2	93	16	17	67	19	7	0

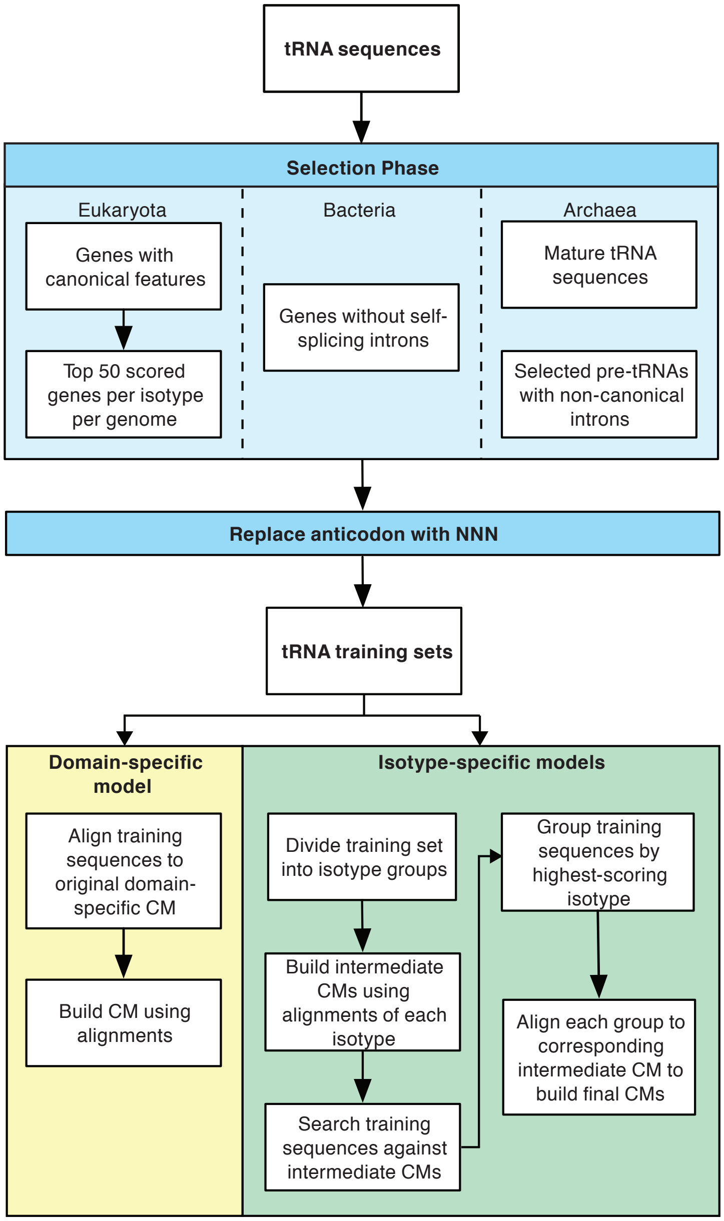

Supplementary Figure S1. Schematic diagram of covariance model generation. A training set for covariance model generation was created for each domain of life. Domain-specific CMs were built for eukaryotes, bacteria, and archaea with two rounds of training to build isotype-specific covariance models.

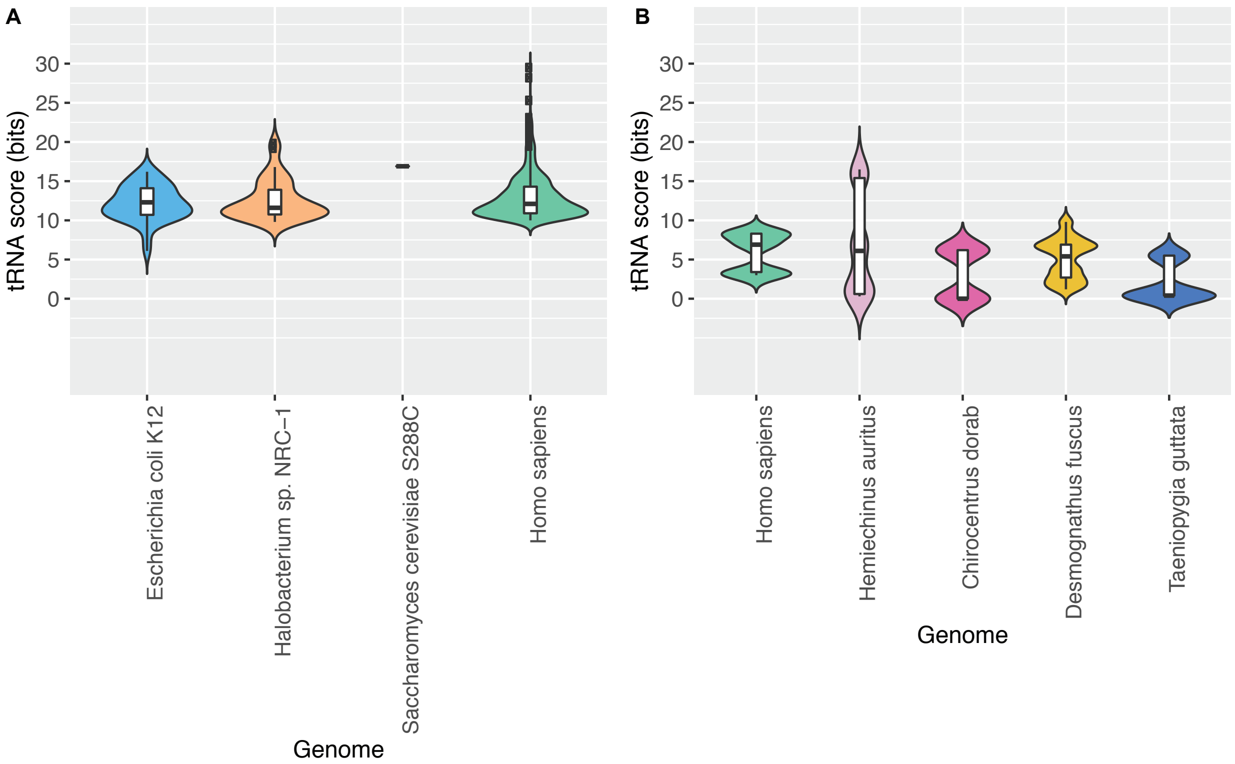

Supplementary Figure S2. tRNAscan-SE 2.0 score prediction distributions to assess false positives in (A) virtual nuclear genomes using corresponding domain search modes, and (B) virtual mitochondrial genomes using vertebrate covariance models. tRNA prediction results are also summarized in Supplementary Tables S3 and S4. Based on these distributions, a default cutoff of 20 bits was not changed from the original v1.3. The boxes inside the violin plot represent the interquartile range while the black bands represent the median of the tRNA scores.

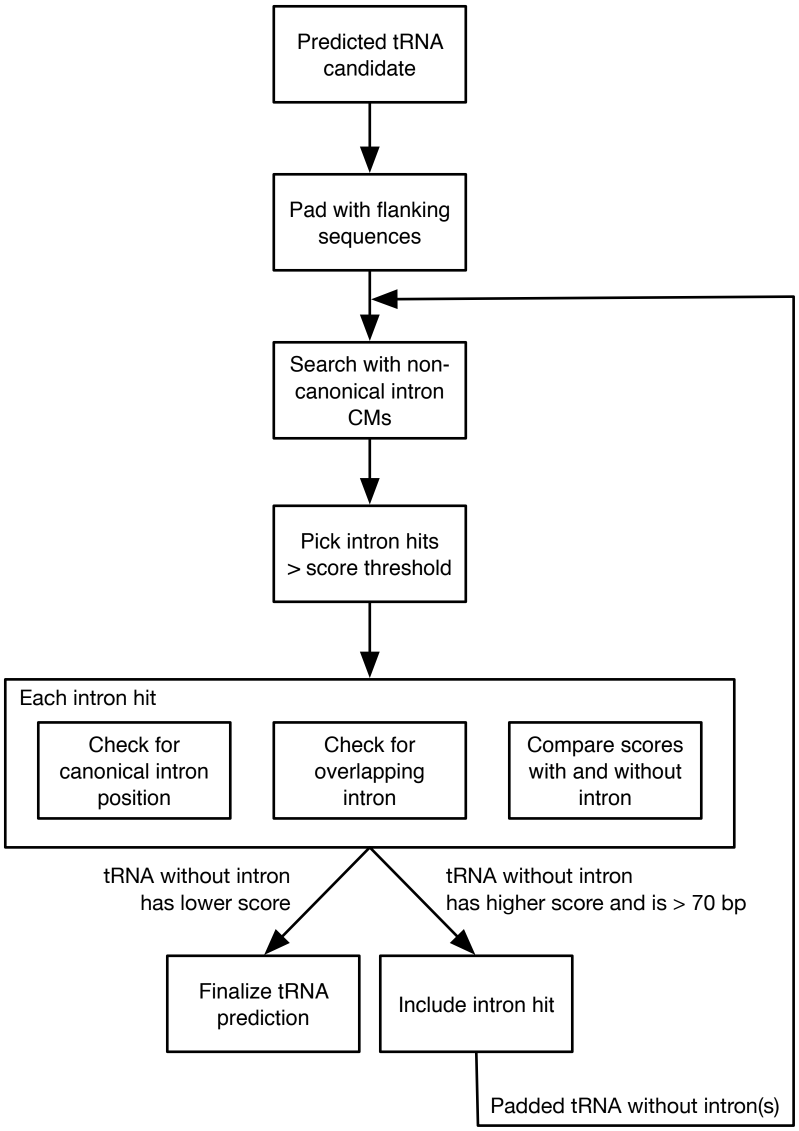

Supplementary Figure S3. Schematic diagram for detecting noncanonical introns in archaeal tRNA genes. The tRNA gene candidates were padded with flanking sequences and scanned with covariance models that were trained with previously identified noncanonical introns in archaeal tRNAs. The detected introns were characterized by their scores and positions. An iterative process was used to enable detection of multiple introns that are located in close proximity and requires the removal of one intron at a time, resulting in formation of the bulge-helix-bulge secondary structure motif of any potentially remaining intron(s).

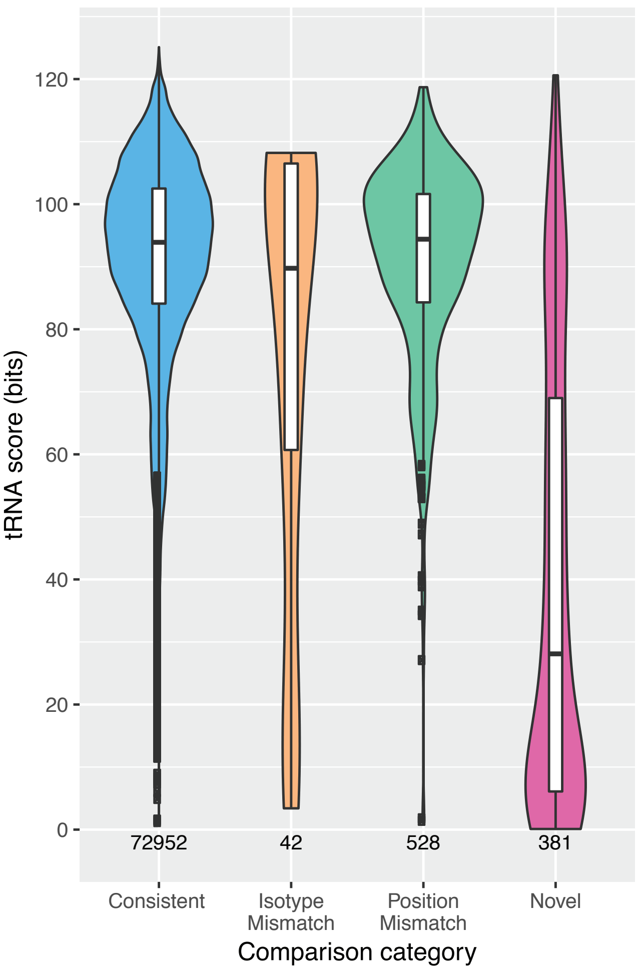

Supplementary Figure S4. Mitochondrial tRNA prediction comparison with NCBI RefSeq (8). 73,903 mt-tRNA genes in 3,345 mitochondrial genomes were predicted using tRNAscan-SE 2.0 with vertebrate mitochondrial search mode and score cutoff of 0 bits. Predictions were compared with 73,674 NCBI RefSeq gene annotations and were grouped into four categories: (1) Consistent – predicted genes are consistent with RefSeq annotations, (2) Isotype Mismatch – predicted genes and RefSeq annotations have different isotype classification, (3) Position Mismatch – predicted genes and RefSeq annotations are on different strands or have different start/end positions but do overlap, and (4) Novel – predicted genes are not annotated in RefSeq. The number below the plot of each category represents the total number of predicted genes for the specified category. The boxes inside the violin plot represent the interquartile range while the black bands represent the median of the tRNA scores.

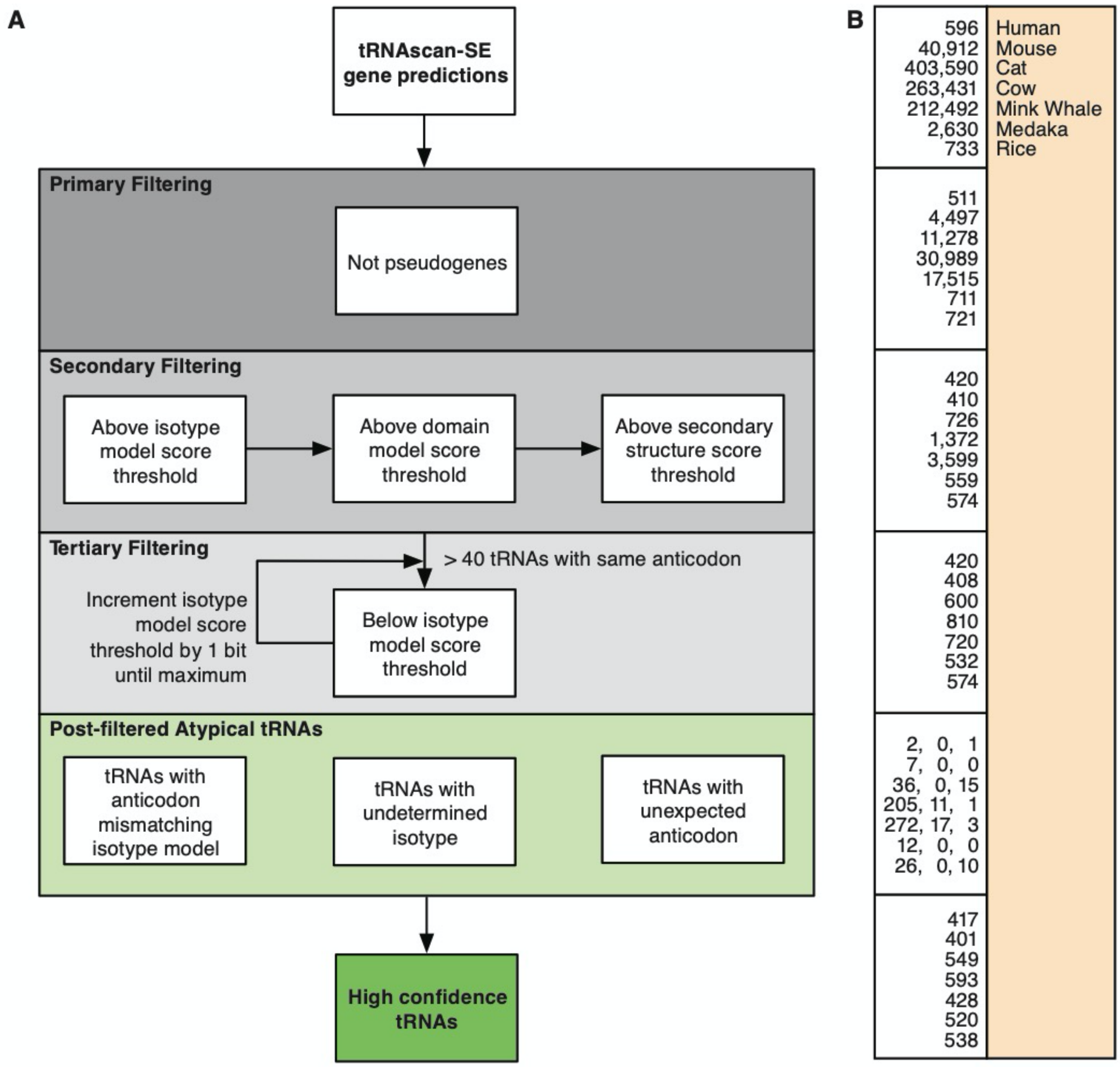

Supplementary Figure S5. Schematic diagram of post-filtering process for identifying high confidence tRNA genes. (A) Flowchart represents the filtering steps used for tRNA predictions in large eukaryotes to better distinguish between tRNA-derived SINEs and real tRNA genes. (B) Counts of tRNA gene predictions of selected genomes are listed at each filtering step.

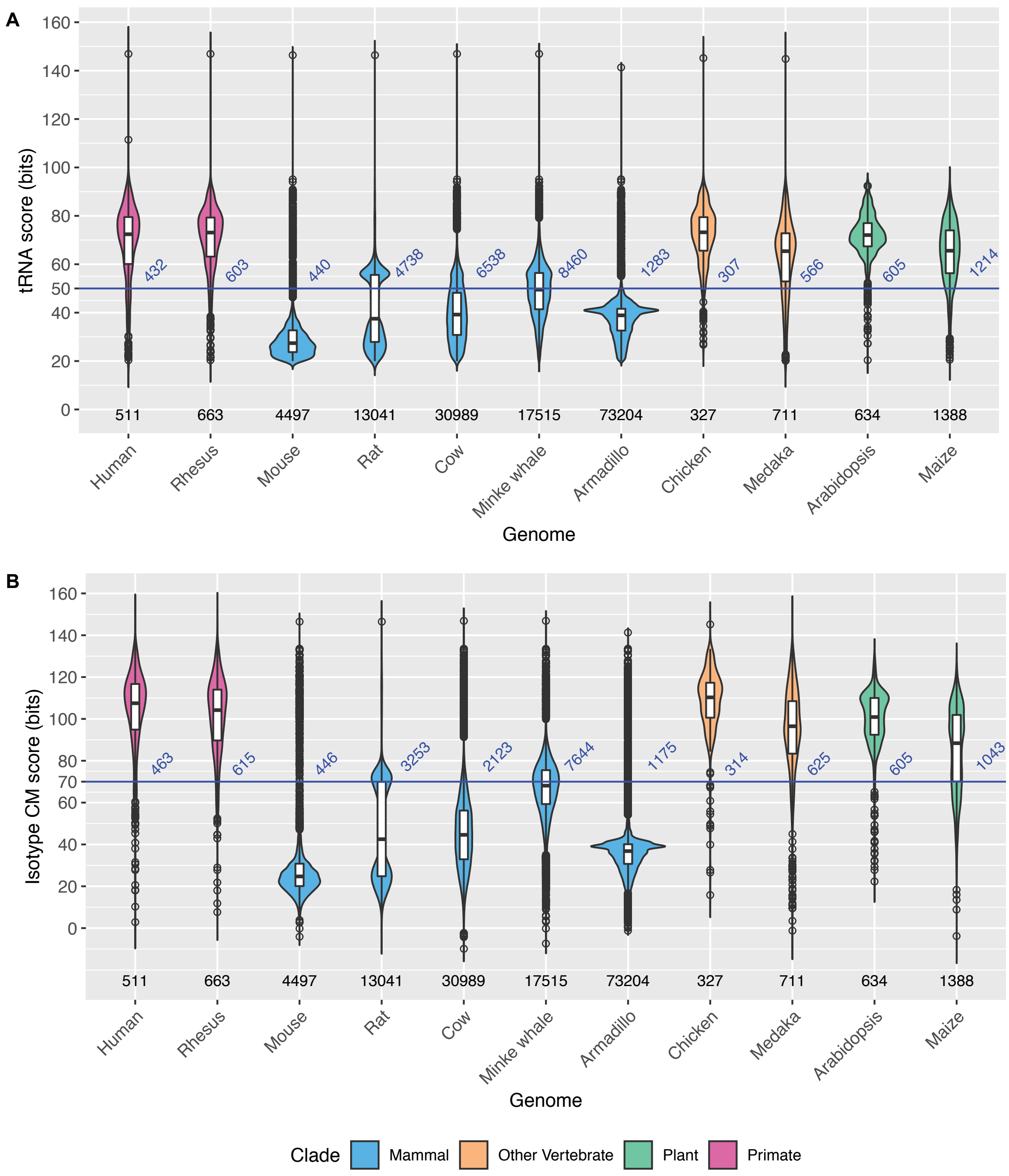

A
tRNA score (bits)
Genome
Human
Rhesus
Mouse
Rat
Cow
Minke whale
Armadillo
Chicken
Medaka
Arabidopsis
Maize
511 663 4497 13041 30989 17515 73204 327 711 634 1388
432 603 440 4738 6538 8460 1283 307 566 605 1214
B
Isotype CM score (bits)
463 615 446 3253 2123 7644 1175 314 625 605 1043
Clade
Mammal
Other Vertebrate
Plant
Primate

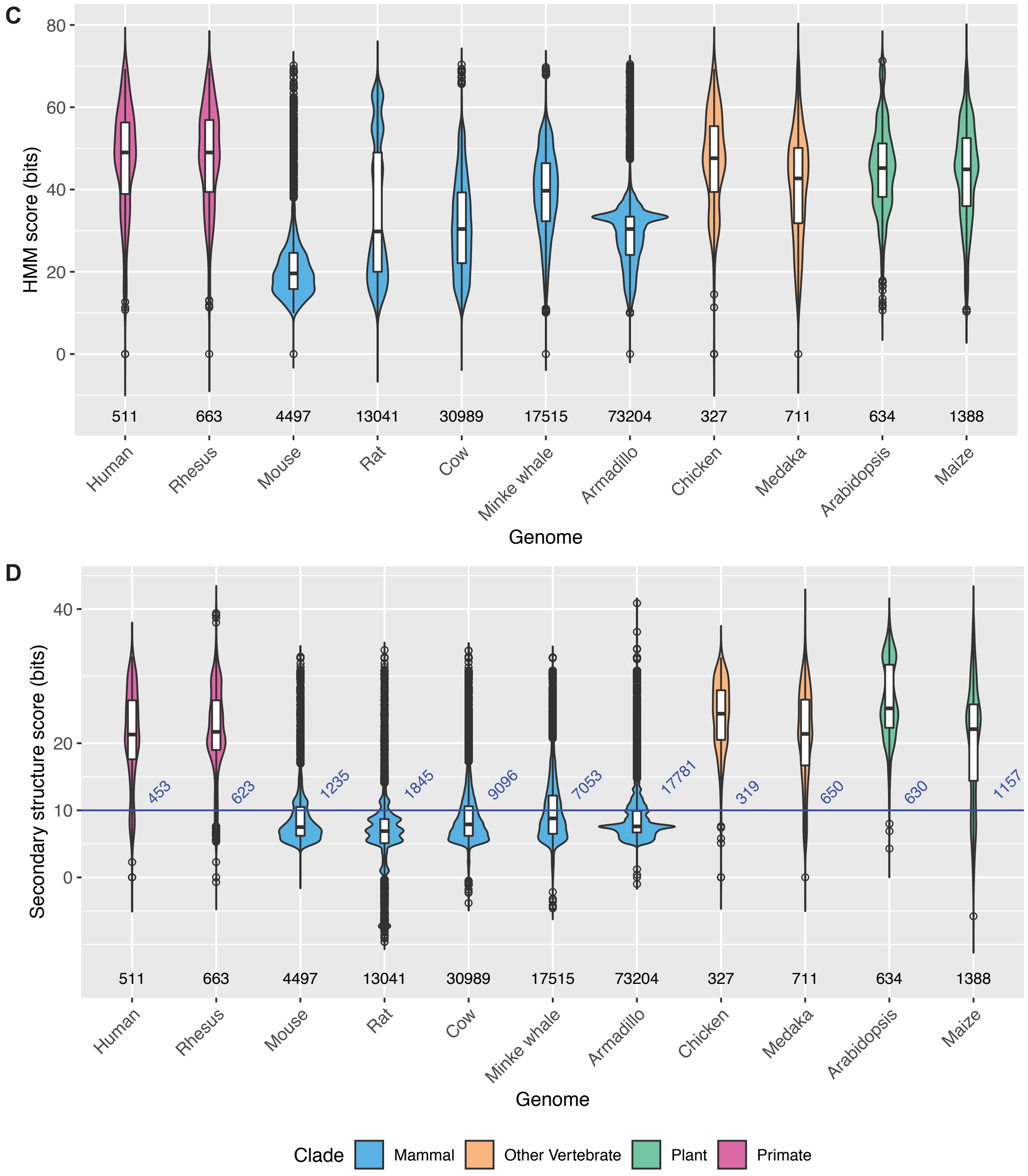

Supplementary Figure S6. tRNA prediction score distributions of selected large eukaryotic genomes, showing a wide range of counts and scores. Predictions classified as pseudogenes have been excluded. Distributions of four score types are included: (A) tRNA (domain-specific) scores, (B) isotype-specific scores, (C) HMM scores (scores based on primary sequences), and (D) secondary structure scores. The horizontal blue lines represent score thresholds used in the post-filtering approach for identifying high confidence tRNA genes. No single filtering threshold is sufficient for removing tRNA-derived repetitive elements for all species, thus a multi-stage filter was developed. The numbers below the violin plots of each genome are the total number of tRNA predictions represented in the distributions. The numbers at the horizontal blue lines are the number of tRNA predictions at the corresponding score thresholds. The boxes inside the violin plot represent the interquartile range while the black bands represent the median of the tRNA scores.

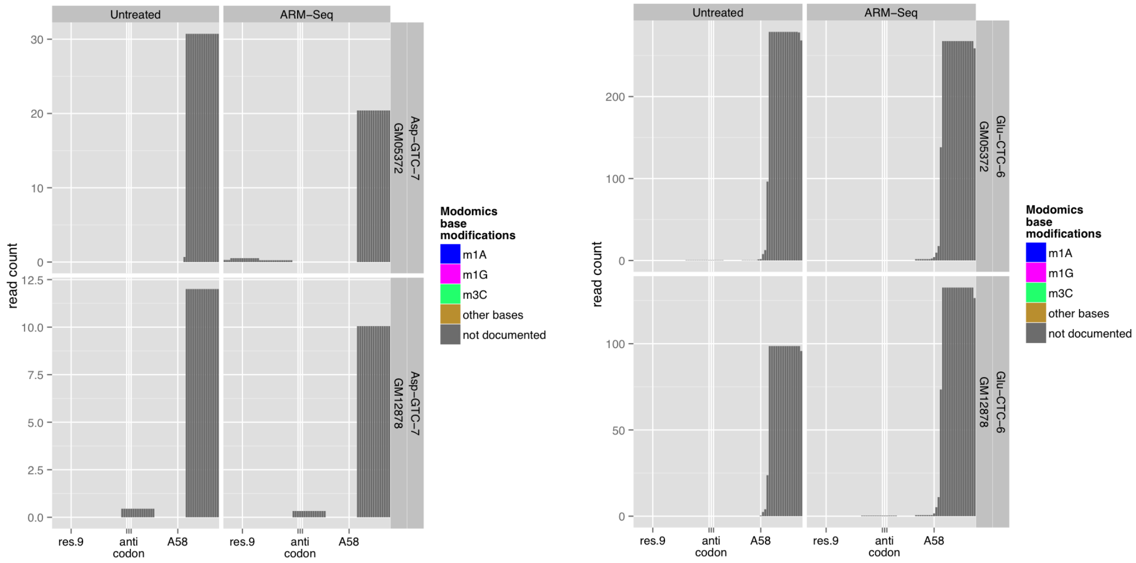

Supplementary Figure S7. Read coverage of tRNA-Asp-GTC-7 and tRNA-Glu-CTC-6 in human from ARM-Seq study (5). Sequencing reads only aligned to the 3' end of the transcripts.

A

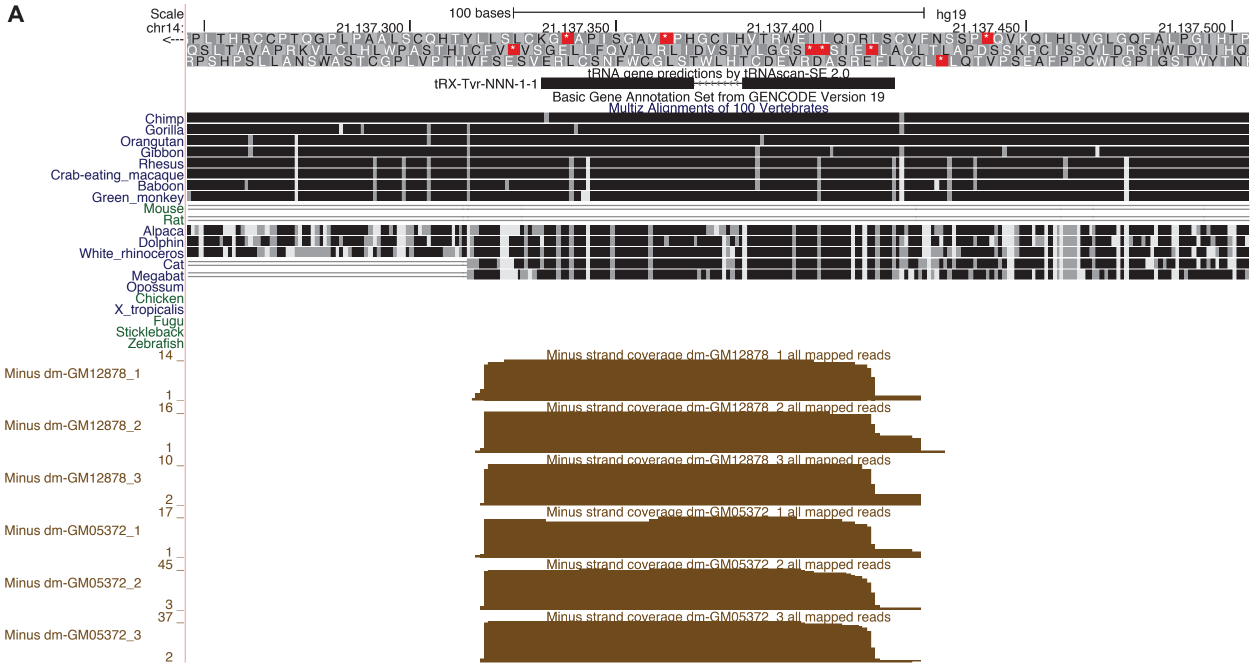

B

Genome	GtRNAdb Gene Symbol	Score (bits)	Feature
Human	tRX-Tyr-NNN-1-1	22.8	Uncertain function
Chimp	N/A	19.1	Below default score cutoff
Gorilla	tRX-Tyr-NNN-3-1	20.7	Uncertain function
Rhesus	tRX-Tyr-NNN-4-1	39.5	Uncertain function
Baboon	tRX-Tyr-NNN-4-1	39.5	Uncertain function
Alpaca	tRNA-Tyr-GTA-3-6	75.4	High confidence
Dolphin	tRNA-Tyr-GTA-6-1	72.9	High confidence
White rhinoceros	tRNA-Tyr-GTA-3-3	75.4	High confidence
Cat	tRNA-Tyr-GTA-3-2	75.4	High confidence

Supplementary Figure S8. tRX-Tyr-NNN-1-1 in human shows uniform expression as a precursor tRNA. (A) The UCSC Genome Browser (9) visualization shows tRX-Tyr-NNN-1-1, a predicted tRNA gene candidate for decoding tyrosine with anticodon GUA. The predicted gene has a 12-nt intron represented by the arrowed portion of the track item and is conserved across primates and some other mammals. The ARM-Seq coverage tracks show uniform coverage across the gene body and the intron instead of higher coverage at the mature sequence region, indicating that the gene transcript may not be processed as a typical tRNA. (B) Orthologs in mammals based on multi-genome alignments are listed with GtRNAdb gene symbols, scores, and feature classification.

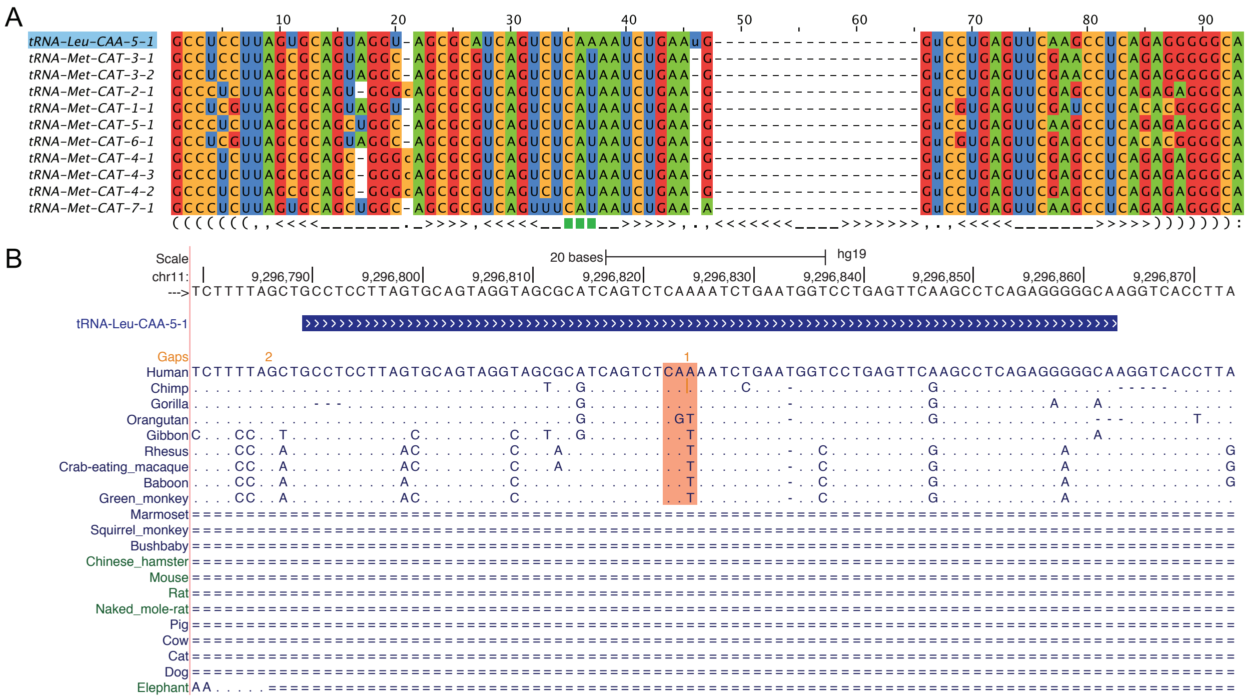

Supplementary Figure S9. Sequence conservation of tRNA-Leu-CAA-5-1 in human. (A) Alignment of tRNA-Leu-CAA-5-1 with all predicted tRNA^{Met} sequences from human shows high conservation supporting its ancestry as a tRNA^{Met} gene. (B) UCSC Genome Browser view of tRNA-Leu-CAA-5-1 locus that is shared with other primates, but not other mammals (“=”). Anticodon CAA (highlighted) is conserved in human, chimp, and gorilla (identity with human reference sequence shown with “.”). The genomes of other primates have anticodon CAT (corresponding to tRNA^{Met}) of this gene instead.

References

1. Nawrocki,E.P. and Eddy,S.R. (2013) Infernal 1.1: 100-fold faster RNA homology searches. *Bioinformatics*, **29**, 2933–5.

2. Steinberg,S., Misch,A. and Sprinzl,M. (1993) Compilation of tRNA sequences and sequences of tRNA genes. *Nucleic Acids Res*, **21**, 3011–5.

3. Eddy,S.R. and Durbin,R. (1994) RNA sequence analysis using covariance models. *Nucleic Acids Res*, **22**, 2079–88.

4. Juhling,F., Putz,J., Bernt,M., Donath,A., Middendorf,M., Florentz,C. and Stadler,P.F. (2012) Improved systematic tRNA gene annotation allows new insights into the evolution of mitochondrial tRNA structures and into the mechanisms of mitochondrial genome rearrangements. *Nucleic Acids Res*, **40**, 2833–45.

5. Cozen,A.E., Quartley,E., Holmes,A.D., Hrabeta-Robinson,E., Phizicky,E.M. and Lowe,T.M. (2015) ARM-seq: AlkB-facilitated RNA methylation sequencing reveals a complex landscape of modified tRNA fragments. *Nat Methods*, **12**, 879–84.

6. Zheng,G., Qin,Y., Clark,W.C., Dai,Q., Yi,C., He,C., Lambowitz,A.M. and Pan,T. (2015) Efficient and quantitative high-throughput tRNA sequencing. *Nat. Methods*, **12**, 835–837.

7. Kuksa,P.P., Amlie-Wolf,A., Katanić,Ž., Valladares,O., Wang,L.-S. and Leung,Y.Y. (2019) DASHR 2.0: integrated database of human small non-coding RNA genes and mature products. *Bioinformatics*, **35**, 1033–1039.

8. O’Leary,N.A., Wright,M.W., Brister,J.R., Ciufo,S., Haddad,D., McVeigh,R., Rajput,B., Robbertse,B., Smith-White,B., Ako-Adjei,D., *et al.* (2016) Reference sequence (RefSeq) database at NCBI: current status, taxonomic expansion, and functional annotation. *Nucleic Acids Res*, **44**, D733-45.

9. Navarro Gonzalez,J., Zweig,A.S., Speir,M.L., Schmelter,D., Rosenbloom,K.R., Raney,B.J., Powell,C.C., Nassar,L.R., Maulding,N.D., Lee,C.M., *et al.* (2021) The UCSC Genome Browser database: 2021 update. *Nucleic Acids Res.*, **49**, D1046–D1057.
