# Supplementary material for: tRNAscan-SE 2.0: improved detection and functional classification of transfer RNA genes
Source: Nucleic Acids Res. 2021 Aug 20;49(16):9077–96. doi: 10.1093/nar/gkab688 (PMC8450103; doi:10.1093/nar/gkab688)
Supplement: gkab688_Supplemental_Files [file gkab688_supplemental_files.zip › tRNAscan-SE_supplementary_file_legends.pdf]

Supplementary File S1 Archaeal tRNA detection result comparison differences between tRNAscan-SE 1.3 and 2.0 (tarball file). Archaea_20_vs_131_diff.txt (tab-delimited text file) is a table representing the tRNA predictions in archaeal genomes that differ in the results generated by v1.3 and v2.0. Archaea_v13_diff.ss (text file) contains the sequences and secondary structures of the corresponding tRNA predictions with differences, found using v1.3. Archaea_v20_diff.ss contains the sequences and secondary structures of the corresponding tRNA predictions with differences, found using v2.0. These are useful to identify the atypical features in tRNAs that caused differences in detection/annotation.

Supplementary File S2 Bacterial tRNA detection result comparison differences between tRNAscan-SE 1.3 and 2.0 (tarball file). Bacteria_20_vs_131_diff.txt (tab-delimited text file) is a table representing the tRNA predictions in bacterial genomes that differ in the results generated by v1.3 and v2.0. Bacteria_v13_diff.ss (text file) contains the sequences and secondary structures of the corresponding tRNA predictions with differences, found using v1.3. Bacteria_v20_diff.ss (text file) contains the sequences and secondary structures of the corresponding tRNA predictions differences, found using v2.0.

Supplementary File S3 Eukaryotic tRNA detection result comparison differences between tRNAscan-SE 1.3 and 2.0 (tarball file). Euk_20_vs_131_diff.txt (tab-delimited text file) is a table representing the tRNA predictions in eukaryotic genomes that differ in the results generated by v1.3 and v2.0. Euk_v13_diff.ss (text file) contains the sequences and secondary structures of the corresponding tRNA predictions with differences, found using v1.3. Euk_v20_diff.ss (text file) contains the sequences and secondary structures of the corresponding tRNA predictions differences, found using v2.0.

Supplementary File S4 tRNA and mitochondrial tRNA predictions for virtual genomes (tarball file). The compressed text files represent the results generated by tRNAscan-SE 2.0 for scanning the virtual genomes listed in Supplementary Tables S4 and S5.

Supplementary File S5 Missing tRNAs with anticodon CAU (Excel file). Genomes that have unidentified tRNAs with CAU anticodon are listed. The “Note” column represents the analysis results for the possible causes of missing tRNA genes.

Supplementary File S6 Isotype inconsistency analysis (Excel file). Percentages of tRNA predictions with differences between the highest-scoring isotype-specific model and the predicted anticodon were computed. The rows in the tables represent the isotypes corresponding to the predicted anticodons while the columns represent the highest-scoring isotype-specific models.

Supplementary File S7 Mitochondrial genomes in RefSeq (Excel file). List of mitochondrial genomes from NCBI RefSeq and RefSeq accession numbers used in mt-tRNA prediction comparison.

Supplementary File S8 Mitochondrial tRNA prediction comparison with RefSeq (tab-delimited text file, compressed). Comparison of the mt-tRNA genes predicted by tRNAscan-SE 2.0 and the annotated mt-tRNAs in the NCBI RefSeq mitochondrial genomes listed in Supplementary File S7. The columns in the file are: (1) genome name/tRNAscan-SE id; (2) tRNA start coordinate, (3) tRNA end coordinate, (4) tRNA strand, (5) tRNA isotype, (6) tRNA anticodon, and (7) tRNA bit score, from tRNAscan-SE results; (8) tRNA start coordinate, (9) tRNA end coordinate, (10) tRNA strand, and (11) tRNA isotype, from RefSeq annotations; (12) comparison result.

Supplementary File S9 Mitochondrial tRNA comparison differences between prediction algorithms and RefSeq (tarball file). arwen_refseq_diff.txt, mitfi_refseq_diff.txt, and tRNAscan-SE-2_refseq_diff.txt (tab-delimited text files) represent the mt-tRNA predictions generated by ARWEN, MiTFi, and tRNAscan-SE 2.0, respectively, which differ from the annotated mt-tRNAs in NCBI RefSeq.
